# Plasma adenosine deaminase-1 and -2 activities are lower at birth in Papua New Guinea than in The Gambia but converge over the first weeks of life

**DOI:** 10.3389/fimmu.2024.1425349

**Published:** 2024-09-25

**Authors:** Thomas S. Kouyate, Athena N. Nguyen, Alec L. Plotkin, Rebeca Ford, Olubukola T. Idoko, Oludare A. Odumade, Geraldine Masiria, Joe Jude, Joann Diray-Arce, Kerry McEnaney, Al Ozonoff, Hanno Steen, Tobias R. Kollmann, Peter C. Richmond, Anita H. J. van den Biggelaar, Beate Kampmann, William Pomat, Ofer Levy, Kinga K. Smolen

**Affiliations:** ^1^ Precision Vaccines Program, Department of Pediatrics, Boston Children’s Hospital, Boston, MA, United States; ^2^ Papua New Guinea Institute of Medical Research, Goroka, Papua New Guinea; ^3^ Vaccines & Immunity Theme, Medical Research Council Unit The Gambia at the London School of Hygiene and Tropical Medicine, Banjul, Gambia; ^4^ The Vaccine Centre, Faculty of Infectious and Tropical Diseases, London School of Hygiene and Tropical Medicine, London, United Kingdom; ^5^ Harvard Medical School, Boston, MA, United States; ^6^ Division of Medicine Critical Care, Boston Children’s Hospital, Boston, MA, United States; ^7^ Broad Institute of Massachusetts Institute of Technology & Harvard, Cambridge, MA, United States; ^8^ Department of Pathology, Boston Children’s Hospital, Boston, MA, United States; ^9^ Telethon Kids Institute, Subiaco, WA, Australia; ^10^ Wesfarmers Centre of Vaccines and Infectious Diseases, Telethon Kids Institute, University of Western Australia, Perth, WA, Australia; ^11^ Division of Pediatrics, School of Medicine, University of Western Australia, Perth Children’s Hospital, Perth, WA, Australia; ^12^ Charité Centre for Global Health and Institute for International Health, Charité – Universitätsmedizin, Berlin, Germany

**Keywords:** adenosine deaminase, ontogeny, global health, infant, immune development

## Abstract

**Introduction:**

Dynamic cellular and molecular adaptations in early life significantly impact health and disease. Upon birth, newborns are immediately challenged by their environment, placing urgent demands on the infant immune system. Adenosine deaminases (ADAs) are enzymatic immune modulators present in two isoforms – ADA-1 and ADA-2. Infants exhibit low ADA activity, resulting in high plasma adenosine concentrations and a consequent anti-inflammatory/anti-Th1 bias. While longitudinal studies of plasma ADA have been conducted in infants in The Gambia (GAM), little is known regarding ADA trajectories in other parts of the world.

**Methods:**

Herein, we characterized plasma ADA activity in an infant cohort in Papua New Guinea (PNG; n=83) and compared to ontogeny of ADA activity in a larger cohort in GAM (n=646). Heparinized peripheral blood samples were collected at day of life (DOL) 0, DOL7, DOL30, and DOL128. Plasma ADA-1, ADA-2, and total ADA activities were measured by chromogenic assay.

**Results:**

Compared to GAM infants, PNG infants had significantly lower ADA-1 (0.9-fold), ADA-2 (0.42-fold), and total ADA (0.84-fold) activities at birth which converged by DOL30.

**Discussion:**

Overall, discovery of a distinct baseline and a consistent pattern of increasing plasma ADA activity in early life in two genetically and geographically distinct populations validates and extends previous findings on the robustness of early life immune ontogeny.

## 1 Introduction

Early life is characterized by robust ontogenetic changes in cellular and molecular pathways that significantly impact health and disease in neonates. Newborns, equipped with a relatively naïve immune system, are immediately challenged by their environment (1–3). While the majority of infants undergo a healthy physiological and immunological development, some experience deviations in developmental trajectories, resulting in clinical pathology such as infections. Human blood plasma is a rich source of age-dependent factors that modulate immunity (4). Characterizing soluble plasma factors that influence healthy human immune system development is essential to understanding early life immune dynamics.

Adenosine deaminases (ADAs) -1 and -2 are soluble immunoregulatory proteins present in human plasma that play key roles in health and disease (5, 6). ADA-1, produced by all cells (7), has an intracellular and extracellular role, and forms complexes with CD26 and A2a receptors to promote T-cell proliferation (8, 9). ADA-1 converts immunosuppressive adenosine to immunologically inert inosine (10), thereby enhancing pro-inflammatory responses and Th1 cytokine production (11–13). ADA-1 deficiency leads to impaired thymocyte development and B-lymphocyte immunoglobulin production, resulting in severe combined immunodeficiency (SCID) (14). Furthermore, lower concentrations of ADA-1 in early life correlate with higher adenosine and reduced inflammatory and Th1 polarizing immune responses (6).

ADA-2 is enzymatically less active than ADA-1. While residual ADA-2 activity is measured in patients with ADA-1 deficiency, its specific role within the immune system is not completely characterized (7). Secreted extracellularly by activated monocytes, macrophages, and dendritic cells (7, 15), ADA-2 inhibits the inflammatory response (16, 17) by binding receptors on leukocytes, such as CD39+ regulatory T-cells or CD16+ monocytes, thereby inducing monocyte differentiation to anti-inflammatory macrophages (16). ADA-2 is of growing interest since the discovery of ADA-2 deficiency (DADA2) in 2014 (18, 19). This disease caused by a mutation in *CERC1*, the gene encoding ADA-2, results in vasculitis and bone marrow failure, as well as more rarely pure red cell aplasia (7, 20, 21).

In one of our previous studies, we characterized the ontogeny of ADA activity in infants in The Gambia (GAM, West Africa), and revealed an increase of ADA-1 and ADA-2 activity across the first four months of life in healthy term African infants (5). Our comprehensive study in the GAM described the ADA activity trajectories in early life in a single population. To assess if the plasma ADA patterns observed in GAM are generalizable, we characterized plasma ADA activity in a geographical and genetically distinct population in Papua New Guinea (PNG, Oceania).

In this study, we characterized plasma ADA-1, ADA-2, and total ADA activities across the first four months of life in a PNG infant cohort. We investigated the potential effect of demographic factors including age, sex, season of birth, maternal age, and gestational age on ADA activity in newborns in PNG. Finally, we compared ADA activity in the PNG infant cohort with our previous published GAM infant cohort (5). Overall, our study shows that activities of plasma ADA-1, ADA-2, and total ADA in infants in PNG increase during the first four months of life. Our findings suggest that, while some factors such as sex, birth season, and maternal age may associate with modest differences in plasma ADA activity, the majority of differences in ADA activity in the first weeks of life were driven by ontogeny. When comparing both cohorts, similar patterns in ADA activity are observed, with initial differences detectable at birth that converge by four months of life. Our work supports the hypothesis of diversity at baseline with converging human immune trajectories in early life.

## 2 Materials and methods

### 2.1 Study design

This study is part of the Expanded Program on Immunization Consortium (EPIC) clinical trial EPIC002 design as previously described (22, 23). In brief, EPIC-002 is designed to define biomarkers of neonatal vaccine immunogenicity and consists of 2 cohorts collected at a) the Medical Research Council (MRC) Unit at the London School of Hygiene and Tropical Medicine site in Banjul, The Gambia (GAM), and b) the Papua New Guinea Institute of Medical Research (PNG-IMR) in Goroka, Papua New Guinea. At both sites, mother-infant dyads were recruited upon delivery. Mothers who delivered vaginally at >37 gestational weeks, were Hepatitis B and human immunodeficiency virus (HIV) negative, and with no history of tuberculosis in the past six weeks (either in the mother or a family member) were eligible for the study. Infants were eligible if they weighed >2.5 kg, had an Apgar score >8 at 5 mins after birth, and had no congenital abnormalities nor infections. The gestational age is reported as estimated based on last menstrual periods or local practice which does not include ultrasound measurements due to lack of availability. Samples were collected across 4 visits (GAM n=720, PNG n=100). Visit 1 samples were collected at DOL0 (within first 24 hours of life); Visit 2 samples were collected at either DOL1, DOL3, or DOL7 at the GAM site, and DOL7 only at the PNG site; Visit 3 samples were collected at DOL30; and Visit 4 samples were collected at DOL128 (~4 months of age). Plasma samples were collected and stored at -80°C prior to batch shipment for storage and subsequent ADA assay.

### 2.2 Adenosine deaminase assay

Plasma ADA-2 and total ADA activity were measured using Adenosine Deaminase Assay Kits [cat. #DZ117A] (Diazyme Laboratories; Poway, CA, USA). Each kit included an ADA calibrator [cat. #DZ117A-Cal], Quality Controls [cat. #DZ117A- Con], and ADA Assay Reagents [cat. #DZ117A]. The plasma samples of participants, ADA calibrator and quality control were loaded onto a Corning CellBIND^®^ 384-well plate in quadruplicate, and 20 μM of Erythro-9-(2-hydroxy-3- nonyl) adenine (EHNA) [cat. #1261] (Tocris Bioscience; Bristol, UK) was pipetted in half of the wells. ADA-1 is inhibited by EHNA, such that the wells containing EHNA are only representative of ADA-2 activity. The remaining wells were loaded with similar volumes of DPBS, which represent total ADA activity in plasma. ADA-1 activity was obtained by taking the difference between the total ADA and ADA-2 activity measured. Following EHNA and DPBS loading, the ADA assay reagents were added. To measure absorbance, each 384-well plates were individually read on an Infinite M Plex (Tecan, Mannedorf, Switzerland) with the absorbance recorded every 5 min for an hour at a wavelength of 550 nm and at 37°C. Plasma ADA activity was obtained by measuring the change in absorbance and averaging the duplicates for individual time points. The average absorbance rate was then converted to ADA activity (in units/liter (U/L)) using a log-standard curve.

### 2.3 Statistical methods

Statistical analysis was performed in R version 4.1.2. The Wilcoxon rank-sum test was employed to compare ADA activity in PNG, in both cohorts, and the demographic factors over time (ggpubr_0.6.0 package). The development of each ADA group activity over the first four months of life in the two cohorts employed an ANOVA test (ggpubr_0.6.0 package). The trajectories of ADA over time in both cohorts were generated using the Locally Estimated Scatterplot Smoothing (LOESS) method (ggplot2_3.4.2 package). The following packages were used for data formatting and visualization: dplyr_1.1.2, tidyr_1.3.0, tidyverse_2.0.0, table1_1.4.3, rstatix_0.7.2, patchwork_1.1.2, grid_4.1.2, gtsummary_1.7.2, and alpacage_0.1.0.

## 3 Results

Out of the 720 participants enrolled in GAM and 100 participants enrolled in PNG, samples from 646 and 83 infants, respectively, were included in the final analysis of this work. As presented in **Table 1**, most mothers were < 35 years old when giving birth (81.9% in PNG and 76.9% in GAM). Two-thirds (67.5%) of mothers at the GAM site reached an estimated full-term pregnancy, defined as >39 to <41 gestational weeks, whereas only 33.7% of mothers in PNG reached an estimated full-term. 66.3% of mothers in PNG delivered at an estimated early term (37 to 38.6 weeks of gestation). Newborns were 53.0% female in PNG and 48.6% female in GAM. The average birth weight was 3,300g in PNG and 3,160g in GAM, and most infants were breastfed at birth (98.8% in PNG and 88.7% in GAM). Most infants in our PNG cohort were born during the wet season (57.8%), while most infants in our Gambian cohort were born during the dry season (66.1%).

**Table 1:** Clinical cohort for participants recruited at the Institute of Medical Research in PNG and Medical Research Council Unit in GAM.

	PNG (N=83)	GAM (N=646)
Maternal Age (years)		
<35	68 (81.9%)	497 (76.9%)
>=35	15 (18.1%)	149 (23.1%)
Gestational Age (weeks)		
Early Term (37-38 wks) Full Term (39-41 wks) Missing	55 (66.3%) 28 (33.7%) 0 (0%)	162 (25.1%) 436 (67.5%) 48 (7.4%)
Sex		
Female	44 (53.0%)	314 (48.6%)
Male	39 (47.0%)	332 (51.4%)
Weight at Birth (gram)		
Mean (SD) Median [Min, Max]	3330 (458) 3330 [2500, 4300]	3160 (383) 3150 [2500, 4400]
Breastfeeding at Birth		
Yes No	82 (98.8%) 1 (1.2%)	575 (88.7%) 73 (11.3%)
Season of Birth^a^		
Dry season Wet season	35 (42.2%) 48 (57.8%)	427 (66,1%) 219 (33.9%)
APGAR Score		
Mean (SD) Median [Mean, Max]	9.87 (0.823) 10.0 [8.00, 15.0]	9.61 (0.587) 10.0 [8.00, 10.0]

**^a^** Dry Season= May-Oct in PNG and Nov-May in GAM; Wet Season= Nov-Apr in PNG and June-Oct in GAM.

### 3.1 Ontogeny of ADA activity in the PNG cohort over the first four months of life

**Figure 1:**
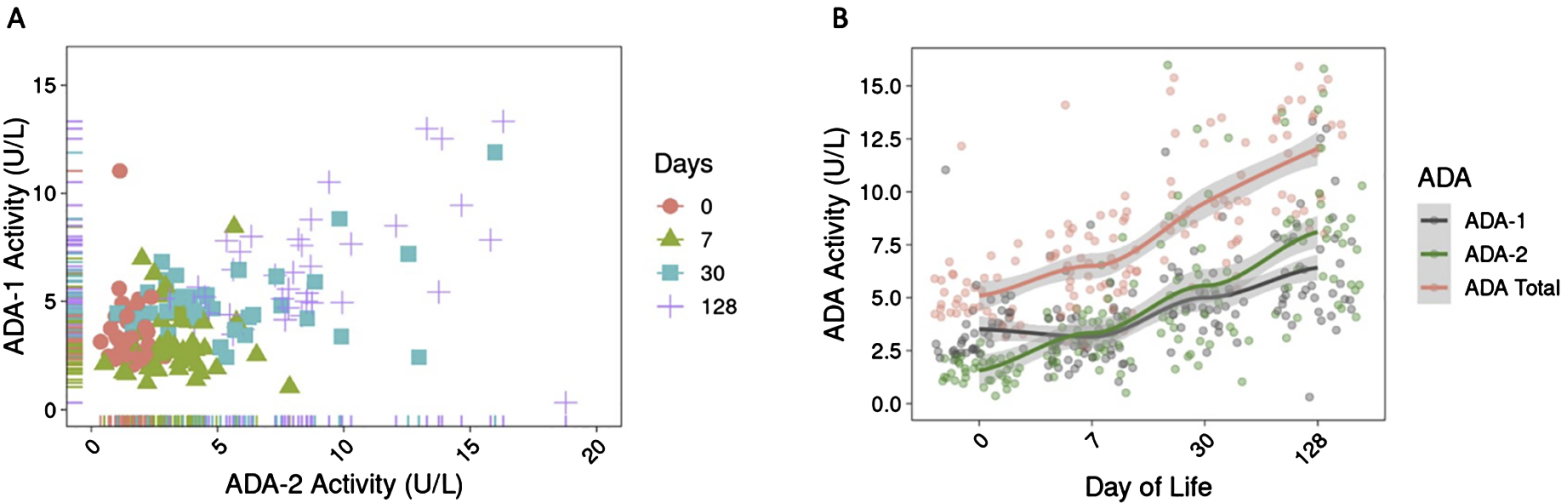
Plasma ADA activity increased over the first four months of life in infants in PNG. **(A)** Activity of ADA-1 and ADA-2 (in U/L) positively correlated over time and are organized by clusters based on day of life, revealing the role of ontogeny on ADA development in early life. **(B)** Activity of ADA-1, ADA-2, and total ADA over the first four months of life showed that ADA activity (in U/L) increased over time. Activity of ADA-1 is higher than ADA-2 at birth but lower at DOL128. The trend lines were generated using LOESS method.

We investigated the effect of ontogeny on plasma ADA activity across the first four months of life in healthy infants in the PNG cohort. The scatterplot of ADA-1 vs. ADA-2 revealed that both isoforms increased over time and fell into clusters based on day of life (DOL) (**Figure 1A**). A similar positive correlation between ADA-1 and ADA-2, as well as age-based groupings were observed in infants in GAM (**Supplementary Figure S1**), highlighting that variation in plasma ADA activity were associated with changes of ontogeny.

Overall, activity of ADA-1, ADA-2, and total ADA significantly increased over the first four months of life (p<2e-16) (**Supplementary Table S1**). Between DOL0 and DOL128, there was a 1.8-fold increase in ADA-1 activity, 5.1-fold increase in ADA-2 activity, and 2.4-fold increase in total ADA activity (**Figure 1B**). No significant increase was observed for ADA-1 during the first week of life (p=0.17) (**Supplementary Table S1**). We also noted that the activity of ADA-1 was greater than that of ADA-2 at DOL0 in infants in PNG but was lower than ADA-2 activity by DOL128 (**Figure 1B**).

### 3.2 ADA activity in PNG appears to be driven by ontogeny

Among the demographic features we measured, few appeared to be associated with higher plasma ADA concentrations. Some differences include 1) male sex: boys had elevated activity of ADA-1 at DOL 30 compared to girls (p=0.039), 2) birth during the dry season (May to October) was associated with higher total ADA activity at DOL 0 than birth during wet season (April to November) (p=0.043), and 3) birth to younger mothers (<35 years old) was associated with a higher total ADA activity at birth compared to birth to older mothers (>35 years old; p=0.031). No other significant differences were identified in association with the other demographic features measured including estimated gestational age at birth (**Supplementary Figure S3** and **Supplementary Tables S2-S4**).

### 3.3 Plasma ADA activities are lower at birth in PNG vs GAM but converge across the first weeks of life

**Figure 2:**
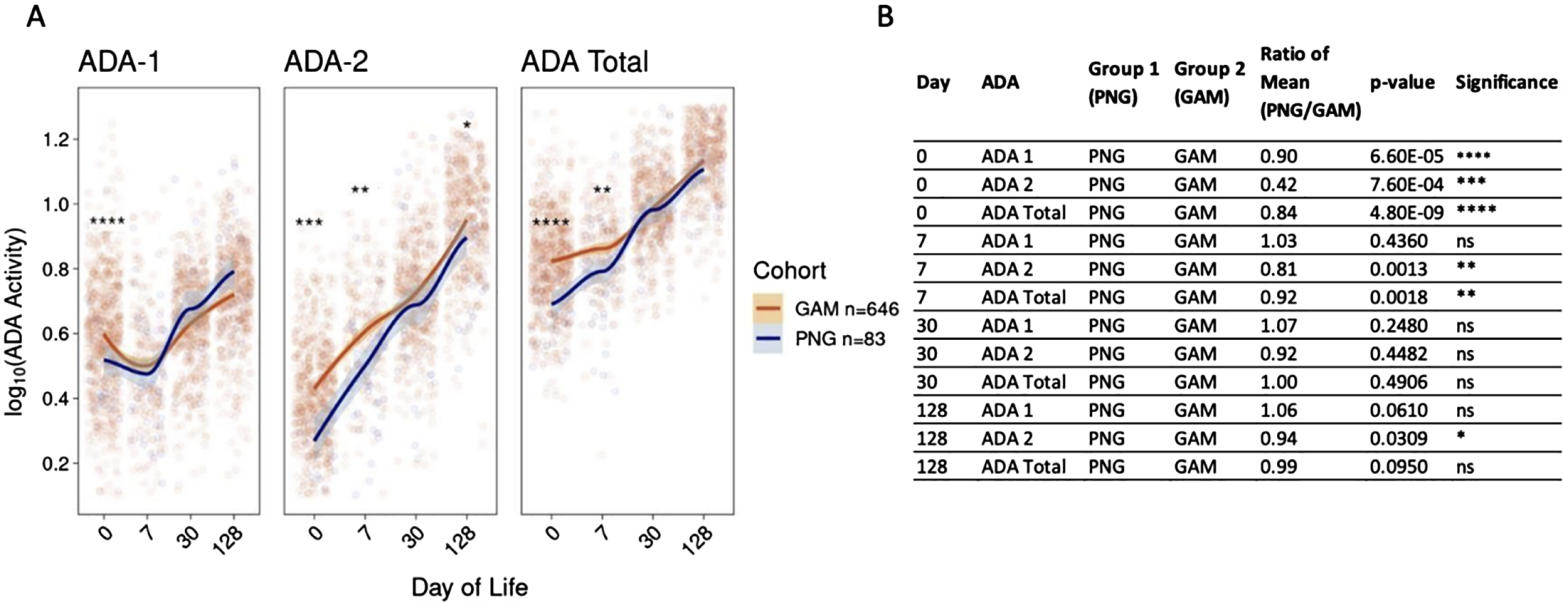
Activity of plasma ADA-1, ADA-2, and total ADA in PNG infants are lower than those of GAM infants in the first week of life then converge by 30 days of life. **(A)** Initial differences between both sites are observed, with infants in PNG having lower ADA-1, ADA-2, and total ADA activity at birth compared to infants in GAM. Activity converged by the first week of life for ADA-1, and by the first month of life for ADA-2 and total ADA. ADA activity was log10 transformed and the trend lines were generated using the LOESS method. **(B)** P-values and mean ratios comparing ADA-1, ADA-2, and total ADA activity of newborns in PNG to those in GAM at DOL0, 7, 30, and 128. Statistical differences were observed at birth, but differences generally decreased over time. A Wilcoxon Rank-sum Test was performed comparing both cohorts, with ns= not significant, *p<0.05, **p<0.01, ***p<0.001, ****p<0.0001.

Comparison of plasma ADA activity over the first four months of life revealed that activity of ADA-1, ADA-2, and total ADA in healthy infants from both PNG and GAM differed at birth but followed a similar pattern that converged over time (**Figure 2A**). A comparison over time followed by an ANOVA test demonstrated differences in ADA-1, ADA-2, and total ADA activity within each individual cohort (**Supplementary Figure S2**). Initial differences in ADA activity were observed between cohorts, with newborns in PNG showing significantly lower activity of ADA-1 (Mean PNG/Mean GAM = 0.90, p=6.6e-5), ADA-2 (Mean PNG/Mean GAM = 0.42, p=7.6e-4), and total ADA (Mean PNG/Mean GAM = 0.84, p=4.8e-9) at birth compared to newborns in GAM (**Figures 2A, B**). By the first week of life, the activity of ADA-1 was similar between the two cohorts (Mean PNG/Mean GAM = 1.03, p = 0.44). PNG study infants still had lower activity of ADA-2 (Mean PNG/Mean GAM = 0.81, p=0.0013) and total ADA (Mean PNG/Mean GAM = 0.92, p=0.0018) compared to those in GAM. However, the ratio of mean was closer to one at DOL7 compared to DOL0, highlighting an already noticeable convergence in ADA activity. At one month of life, no significant differences in ADA activity were noticeable between cohorts, and at four months of age, only a slight difference in ADA-2 was observed with infants in PNG having a lower activity compared to infants in GAM (Mean PNG/Mean GAM = 0.94, p=0.0309).

## 4 Discussion

Herein, we provide the first comparison of plasma ADA activity across the first weeks of life in two genetically and geographically distinct unique longitudinal infant cohorts. We found that the activity of plasma ADA-1, ADA-2, and total ADA in a PNG infant cohort is driven by ontogeny, as observed by the significant increases over the first four months of life. Moreover, comparison of PNG to GAM cohorts revealed that PNG infants demonstrated lower ADA activity in the first week of life compared to GAM infants; however, differences were no longer apparent by one month of age. Thus, the PNG cohort confirms our previous findings on ADA activity in GAM infants and elucidates a robust trajectory of ADA activity across different populations.

The infant immune system undergoes important age-dependent changes in early life (24–27). The dynamic cellular and molecular changes follow a developmental trajectory, as illustrated with immunological changes (28), such as the switch from a Th2-polarized cytokine production to a more balanced Th1/Th2 immunity (29). Relative deficiency of ADA-1 in early life corresponds to high plasma adenosine concentrations and thereby contributes to Th2 polarization and impaired cellular immunity (6). The increasing levels of ADA-1 across the first weeks and months of life noted in this study may contribute to a gradual acquisition of Th1-polarized immunity by late infancy. ADA-2 can promote monocyte differentiation to anti-inflammatory macrophages and CD4+ T cell proliferation (8, 30–32). However, despite the growing interest in ADAs (33–35), the functional role of ADA-2 in infants is not fully understood, and further research is needed to characterize its contribution to the maturation of the infant immune system.

Comparison of plasma ADA-1, ADA-2, and total ADA activity in the PNG cohort compared to the GAM cohort demonstrated early differences, with PNG study infants expressing lower ADA activity in the first week of life, followed by convergence by one month of age. The two study populations live in distinct regions of the world. Infants in the GAM cohort were born in Banjul, a coastal city located in The Gambia, West Africa, while infants in the PNG cohort were born in Goroka in the highlands of PNG, Oceania. Among demographic factors captured in this study, ontogeny appears to be the primary driver of changes in plasma ADA in these infants born in different highly endemic settings. The observation that gestational age difference between 37-41 weeks did not significantly affect plasma ADA levels is not surprising as a prior systems biology study evaluating multiple immune cell development in preterm versus term infants demonstrated early divergence followed by convergence by 3 months of age (36). Multiple environmental factors such as nutrition, gut microbiome, socioeconomic status, stress, or maternal lifestyle can affect the immune development in early life (36–44). Elucidating which of these mechanisms account for the early life differences between PNG and the GAM cohort noted requires further investigations which should include confirming that ADA developmental trajectories are similar between infants born in pathogen low and non-endemic settings, such as the United States.

Although newborns in PNG had lower plasma ADA activity than GAM newborns, ADA activity converged over time. Similar plasma activity of ADA-1 was observed by one week of age, and differences were not statistically significant after 30 days of life. Convergence of inflammatory and immune pathways has previously been demonstrated in neonatal immunity. In a previous multi-omics analysis focusing on cellular and molecular ontogeny across the first week of life in these two cohorts, we found that infants’ immune systems develop in a similar pattern (28), although ADA was not included in these analyses. Here, we report that after four weeks of life, healthy term infants born in different parts of the world reach similar levels of plasma ADA activity, demonstrating convergence in expression of plasma ADAs. This international multi-site study increases our knowledge of immune development in early life. It reveals the need for future investigations of the immune development in different geographical regions and its response to challenge by infections or immune stimuli such as vaccines.

About 15% of newborns diagnosed with SCID have ADA deficiency as a basis for this condition- i.e. ADA-SCID (45). The occurrence of SCID in newborns is rare [~1:60,000 in regions without inbreeding; ~1:2,000 in regions with inbreeding (46)]. Current screening methods include a T-cell Recombination Excision Circle (TREC) assay from dried blood spots quantified by qPCR (47). Neither The Gambia nor PNG employ routine SCID screening and thus no SCID screenings were performed in our study participants. This is in part due to the fact that newborn screening is limited in most African countries in general (48). However, our study focused on soluble plasma ADA1 and ADA2 which to our knowledge is not an assay used for screening. Of note, a rapid and cost-effective mass spectrometry-based screening method measuring plasma adenosine and deoxyadenosine in dried blood spots may detect ADA-SCID (49). Implementation of a cost-effective screen for ADA-SCID, combined with a second-tier test to reduce false positives (49), could improve routine newborn screening and thereby newborn health.

Our study features several strengths, including (a) comparable design, as the exact same protocol were used in both sites, (b) robust sample sizes, in contrast to many systems immunology studies limited by small sample sizes (36, 50, 51), (c) longitudinal design enabling comparison of each study participants across time including their birth timepoint, and (d) rigorous capture of clinical data.

As with any research effort, our study also has limitations. While sample deterioration was a theoretical risk, we followed a standard operating procedure (SOP) to ensure a cold chain was maintained, samples were sorted and transported at -80C. The samples were analyzed within a year of collection. Our comparison involved a sample size difference between the PNG (n = 83) and GAM (n = 646) cohorts. Despite a smaller PNG cohort, we were able to detect differences in baseline ADA concentrations and validate the pattern in plasma ADA-1, ADA-2, and total ADA activity over the first four months of life. Additionally, while we did not measure the functional consequences of differences in plasma ADA concentrations, we infer their importance based on extensive prior literature (52–54). We were unable to consistently collect maternal plasma ADA concentrations, smoking status, or nutrition of the mother and child (apart from breastfeeding) across the two cohorts. Future studies of maternal and infant plasma ADA dynamics should include pregnancy data and maternal factors for a comprehensive overview.

To our knowledge, this study is among the largest longitudinally sampled ADA studies in infants. It validates previous findings describing ontogeny as the main driver of ADA activity across the first four months of life (5). Comparing both cohorts revealed that levels of ADA-1, ADA-2, and total ADA activity in PNG infants were initially lower than those in GAM but converged by one month of age.

For future studies, it will be important to determine if the development of plasma ADA activity in infants is congruent over an extended period at multiple geographic sites including pathogen low and non-endemic settings. It is essential to note that ADA is only one component of a highly complex immune system. Understanding other parameters will be essential in understanding any functional differences between populations. Additionally, investigating whether the immune system of healthy infants converges more rapidly than that of infants with pathology would provide valuable insights into the long-term dynamics of immune system development in early life in relation to health and disease.

## The expanded program on immunization consortium

Nelly Amenyogbe, Asimenia Angelidou, Winnie Bao, Rym Ben-Othman, Tue B. Bennike, Travis M. Blimke, Morten Bjerregaard-Andersen, Ryan R. Brinkman, Byron Brook, Kendyll Burnell, Bing Cai, Abhinav Checkervarty, Jing Chen, Virginia Chen, Mitchell Cooney, Momoudou Cox, Alansana Darboe, Bhavjinder K. Dhillon, Tida Dibassey, Joann Diray-Arce, Reza Falsafi, Benoit Fatou, Rebecca Ford, Freddy Francis, Christian N. Golding, Robert E.W. Hancock, Danny J. Harbeson, Daniel He, Samuel H. Hinshaw, Annmarie Hoch, Joe Huang, Olubukola T. Idoko, Abdulazeez Imam, Beate Kampmann, Wendy Kirarock, Tobias R. Kollmann, Ken Kraft, Kristina Lindberg Larsen, Jessica Lasky-Su, Amy H. Lee, Ofer Levy, Aaron Liu, Mark Liu, Mehrnoush Malek, Arnaud Marchant, Geraldine Masiria, David Jim, John Paul Matlam, Kerry McEnaney, Caitlyn McLoughlin, Sebastiano Montante, Elena Morrocchi, Jorjoh Ndure, Athena N. Nguyen, Jainaba Njie-Jobe, Oludare A. Odumade, Al Ozonoff, Jensen Pak, Paolo Palma, Edward P. K. Parker, Matthew A. Pettengill, Alec L. Plotkin, William S. Pomat, Shun Rao, Peter C. Richmond, Elishia Roberts, Gerard Saleu, Lilica Sanca, Guzman Sanchez-Schmitz, Frederik Schaltz-Buchholzer, Casey P Shannon, Amrit Singh, Maren Smith, Kinga K. Smolen, Hanno Steen, Julia Strandmark, Caitlin Syphurs, Scott J. Tebbutt, Anita H.J. van den Biggelaar, Simon D. van Haren, Natallia Varankovich, Sofia Vignolo, Diana Vo, Oghenebrume Wariri.

## Data Availability

The datasets presented in this study can be found in online repositories. The names of the repository/repositories and accession number(s) can be found below: The dataset supporting the findings of this study has been deposited in the ImmPort database, accessible under accession number SDY1538.

## References

[1] Black RE Morris SS Bryce J. Where and why are 10 million children dying every year? Lancet. (2003) 361:2226–34. doi: 10.1016/S0140-6736(03)13779-8 12842379

[2] Kollmann TR Kampmann B Mazmanian SK Marchant A Levy O. Protecting the newborn and young infant from infectious diseases: lessons from immune ontogeny. Immunity. (2017) 46:350–63. doi: 10.1016/j.immuni.2017.03.009 28329702

[3] Dowling DJ Levy O. Ontogeny of early life immunity. Trends Immunol. (2014) 35:299–310. doi: 10.1016/j.it.2014.04.007 24880460 PMC4109609

[4] Pettengill MA van Haren SD Levy O. Soluble mediators regulating immunity in early life. Front Immunol. (2014) 5:457. doi: 10.3389/fimmu.2014.00457 25309541 PMC4173950

[5] Odumade OA Plotkin AL Pak J Idoko OT Pettengill MA Kollmann TR. Plasma adenosine deaminase (ADA)-1 and -2 demonstrate robust ontogeny across the first four months of human life. Front Immunol. (2021) 12:578700. doi: 10.3389/fimmu.2021.578700 34122398 PMC8190399

[6] Pettengill M Robson S Tresenriter M Millan JL Usheva A Bingham T. Soluble ecto-5’-nucleotidase (5’-NT), alkaline phosphatase, and adenosine deaminase (ADA1) activities in neonatal blood favor elevated extracellular adenosine. J Biol Chem. (2013) 288:27315–26. doi: 10.1074/jbc.M113.484212 PMC3779727 23897810

[7] Meyts I Aksentijevich I. Deficiency of adenosine deaminase 2 (DADA2): updates on the phenotype, genetics, pathogenesis, and treatment. J Clin Immunol. (2018) 38:569–78. doi: 10.1007/s10875-018-0525-8 PMC6061100 29951947

[8] Zavialov AV Gracia E Glaichenhaus N Franco R Zavialov AV Lauvau G. Human adenosine deaminase 2 induces differentiation of monocytes into macrophages and stimulates proliferation of T helper cells and macrophages. J Leukoc Biol. (2010) 88:279–90. doi: 10.1189/jlb.1109764 20453107

[9] Moreno E Canet J Gracia E Lluis C Mallol J Canela EI. Molecular evidence of adenosine deaminase linking adenosine A(2A) receptor and CD26 proteins. Front Pharmacol. (2018) 9:106. doi: 10.3389/fphar.2018.00106 29497379 PMC5818423

[10] Chen W Hoerter J Gueron MA. comparison of AMP degradation in the perfused rat heart during 2-deoxy-D-glucose perfusion and anoxia. Part I: The release of adenosine and inosine. J Mol Cell Cardiol. (1996) 28:2163–74. doi: 10.1006/jmcc.1996.0208 8930811

[11] Levy O Coughlin M Cronstein BN Roy RM Desai A Wessels MR. The adenosine system selectively inhibits TLR-mediated TNF-alpha production in the human newborn. J Immunol. (2006) 177:1956–66. doi: 10.4049/jimmunol.177.3.1956 PMC2881468 16849509

[12] Bouma MG Jeunhomme TM Boyle DL Dentener MA Voitenok NN van den Wildenberg FA. Adenosine inhibits neutrophil degranulation in activated human whole blood: involvement of adenosine A2 and A3 receptors. J Immunol. (1997) 158:5400–8. doi: 10.4049/jimmunol.158.11.5400 9164961

[13] Borea PA Gessi S Merighi S Vincenzi F Varani K. Pharmacology of adenosine receptors: the state of the art. Physiol Rev. (2018) 98:1591–625. doi: 10.1152/physrev.00049.2017 29848236

[14] Sauer AV Brigida I Carriglio N Aiuti A. Autoimmune dysregulation and purine metabolism in adenosine deaminase deficiency. Front Immunol. (2012) 3:265. doi: 10.3389/fimmu.2012.00265 22969765 PMC3427915

[15] Moens L Hershfield M Arts K Aksentijevich I Meyts I. Human adenosine deaminase 2 deficiency: A multi-faceted inborn error of immunity. Immunol Rev. (2019) 287:62–72. doi: 10.1111/imr.12722 30565235

[16] Kaljas Y Liu C Skaldin M Wu C Zhou Q Lu Y. Human adenosine deaminases ADA1 and ADA2 bind to different subsets of immune cells. Cell Mol Life Sci. (2017) 74:555–70. doi: 10.1007/s00018-016-2357-0 PMC11107696 27663683

[17] Zavialov AV Engstrom A. Human ADA2 belongs to a new family of growth factors with adenosine deaminase activity. Biochem J. (2005) 391:51–7. doi: 10.1042/BJ20050683 PMC1237138 15926889

[18] Zhou Q Yang D Ombrello AK Zavialov AV Toro C Zavialov AV. Early-onset stroke and vasculopathy associated with mutations in ADA2. N Engl J Med. (2014) 370:911–20. doi: 10.1056/NEJMoa1307361 PMC4193683 24552284

[19] Navon Elkan P Pierce SB Segel R Walsh T Barash J Padeh S. Mutant adenosine deaminase 2 in a polyarteritis nodosa vasculopathy. N Engl J Med. (2014) 370:921–31. doi: 10.1056/NEJMoa1307362 24552285

[20] Sharma A Naidu G Sharma V Jha S Dhooria A Dhir V. Deficiency of adenosine deaminase 2 in adults and children: experience from India. Arthritis Rheumatol. (2021) 73:276–85. doi: 10.1002/art.41500 PMC7902299 32892503

[21] Barzaghi F Minniti F Mauro M Bortoli M Balter R Bonetti E. ALPS-like phenotype caused by ADA2 deficiency rescued by allogeneic hematopoietic stem cell transplantation. Front Immunol. (2018) 9:2767. doi: 10.3389/fimmu.2018.02767 30692987 PMC6339927

[22] Idoko OT Smolen KK Wariri O Imam A Shannon CP Dibassey T. Clinical protocol for a longitudinal cohort study employing systems biology to identify markers of vaccine immunogenicity in newborn infants in the Gambia and papua new Guinea. Front Pediatr. (2020) 8:197. doi: 10.3389/fped.2020.00197 32426309 PMC7205022

[23] Idoko OT Smolen KK Wariri O Imam A Shannon CP Dibassey T. Corrigendum: clinical protocol for a longitudinal cohort study employing systems biology to identify markers of vaccine immunogenicity in newborn infants in the Gambia and papua new Guinea. Front Pediatr. (2020) 8:610461. doi: 10.3389/fped.2020.610461 33313031 PMC7707081

[24] Siegrist CA. The challenges of vaccine responses in early life: selected examples. J Comp Pathol. (2007) 137 Suppl 1:S4–9. doi: 10.1016/j.jcpa.2007.04.004 17559867

[25] Basha S Surendran N Pichichero M. Immune responses in neonates. Expert Rev Clin Immunol. (2014) 10:1171–84. doi: 10.1586/1744666X.2014.942288 PMC4407563 25088080

[26] Georgountzou A Papadopoulos NG. Postnatal innate immune development: from birth to adulthood. Front Immunol. (2017) 8:957. doi: 10.3389/fimmu.2017.00957 28848557 PMC5554489

[27] Kollmann TR Levy O Montgomery RR Goriely S. Innate immune function by Toll-like receptors: distinct responses in newborns and the elderly. Immunity. (2012) 37:771–83. doi: 10.1016/j.immuni.2012.10.014 PMC3538030 23159225

[28] Lee AH Shannon CP Amenyogbe N Bennike TB Diray-Arce J Idoko OT. Dynamic molecular changes during the first week of human life follow a robust developmental trajectory. Nat Commun. (2019) 10:1092. doi: 10.1038/s41467-019-08794-x 30862783 PMC6414553

[29] Zaghouani H Hoeman CM Adkins B. Neonatal immunity: faulty T-helpers and the shortcomings of dendritic cells. Trends Immunol. (2009) 30:585–91. doi: 10.1016/j.it.2009.09.002 PMC2787701 19846341

[30] Caorsi R Penco F Schena F Gattorno M. Monogenic polyarteritis: the lesson of ADA2 deficiency. Pediatr Rheumatol Online J. (2016) 14:51. doi: 10.1186/s12969-016-0111-7 27609179 PMC5015262

[31] Kutryb-Zajac B Harasim G Jedrzejewska A Krol O Braczko A Jablonska P. Macrophage-derived adenosine deaminase 2 correlates with M2 macrophage phenotype in triple negative breast cancer. Int J Mol Sci. (2021) 22. doi: 10.3390/ijms22073764 PMC8038600 33916440

[32] Zavialov AV Yu X Spillmann D Lauvau G Zavialov AV. Structural basis for the growth factor activity of human adenosine deaminase ADA2. J Biol Chem. (2010) 285:12367–77. doi: 10.1074/jbc.M109.083527 PMC2852975 20147294

[33] Whitmore KV Gaspar HB. Adenosine deaminase deficiency - more than just an immunodeficiency. Front Immunol. (2016) 7:314. doi: 10.3389/fimmu.2016.00314 27579027 PMC4985714

[34] Signa S Bertoni A Penco F Caorsi R Cafaro A Cangemi G. Adenosine deaminase 2 deficiency (DADA2): A crosstalk between innate and adaptive immunity. Front Immunol. (2022) 13:935957. doi: 10.3389/fimmu.2022.935957 35898506 PMC9309328

[35] Flinn AM Gennery AR. Adenosine deaminase deficiency: a review. Orphanet J Rare Dis. (2018) 13:65. doi: 10.1186/s13023-018-0807-5 29690908 PMC5916829

[36] Olin A Henckel E Chen Y Lakshmikanth T Pou C Mikes J. Stereotypic immune system development in newborn children. Cell. (2018) 174:1277–1292 e1214. doi: 10.1016/j.cell.2018.06.045 30142345 PMC6108833

[37] Brodin P Jojic V Gao T Bhattacharya S Angel CJ Furman D. Variation in the human immune system is largely driven by non-heritable influences. Cell. (2015) 160:37–47. doi: 10.1016/j.cell.2014.12.020 25594173 PMC4302727

[38] Collado MC Cernada M Bauerl C Vento M Perez-Martinez G. Microbial ecology and host-microbiota interactions during early life stages. Gut Microbes. (2012) 3:352–65. doi: 10.4161/gmic.21215 PMC3463493 22743759

[39] Chahal N McLain AC Ghassabian A Michels KA Bell EM Lawrence DA. Maternal smoking and newborn cytokine and immunoglobulin levels. Nicotine Tob Res. (2017) 19:789–96. doi: 10.1093/ntr/ntw324 PMC5939663 28011791

[40] Sjogren YM Tomicic S Lundberg A Bottcher MF Bjorksten B Sverremark-Ekstrom E. Influence of early gut microbiota on the maturation of childhood mucosal and systemic immune responses. Clin Exp Allergy. (2009) 39:1842–51. doi: 10.1111/j.1365-2222.2009.03326.x 19735274

[41] Yatsunenko T Rey FE Manary MJ Trehan I Dominguez-Bello MG Contreras M. Human gut microbiome viewed across age and geography. Nature. (2012) 486:222–7. doi: 10.1038/nature11053 PMC3376388 22699611

[42] Bokulich NA Chung J Battaglia T Henderson N Jay M Li H. Antibiotics, birth mode, and diet shape microbiome maturation during early life. Sci Transl Med. (2016) 8:343ra382. doi: 10.1126/scitranslmed.aad7121 PMC5308924 27306664

[43] Graham JE Christian LM Kiecolt-Glaser JK. Stress, age, and immune function: toward a lifespan approach. J Behav Med. (2006) 29:389–400. doi: 10.1007/s10865-006-9057-4 16715331 PMC2805089

[44] Azad MB Lissitsyn Y Miller GE Becker AB HayGlass KT Kozyrskyj AL. Influence of socioeconomic status trajectories on innate immune responsiveness in children. PloS One. (2012) 7:e38669. doi: 10.1371/journal.pone.0038669 22685596 PMC3369855

[45] Hershfield MS. Genotype is an important determinant of phenotype in adenosine deaminase deficiency. Curr Opin Immunol. (2003) 15:571–7. doi: 10.1016/s0952-7915(03)00104-3 14499267

[46] National Institutes of Health. Screening newborns for deadly immune disease saves lives (2023). Available online at: https://www.nih.gov/news-events/news-releases/screening-newborns-deadly-immune-disease-saves-lives (Accessed July 12, 2024).

[47] Biggs CM Haddad E Issekutz TB Roifman CM Turvey SE. Newborn screening for severe combined immunodeficiency: a primer for clinicians. CMAJ. (2017) 189:E1551–7. doi: 10.1503/cmaj.170561 PMC5738248 29255099

[48] Therrell BL Jr. Lloyd-Puryear MA Ohene-Frempong K Ware RE Padilla CD Ambrose EE. Empowering newborn screening programs in African countries through establishment of an international collaborative effort. J Community Genet. (2020) 11:253–68. doi: 10.1007/s12687-020-00463-7 PMC7295888 32415570

[49] la Marca G Giocaliere E Malvagia S Funghini S Ombrone D Della Bona ML. The inclusion of ADA-SCID in expanded newborn screening by tandem mass spectrometry. J Pharm BioMed Anal. (2014) 88:201–6. doi: 10.1016/j.jpba.2013.08.044 24076575

[50] Smolen KK Ruck CE Fortuno ES 3rd Ho K Dimitriu P Mohn WW. Pattern recognition receptor-mediated cytokine response in infants across 4 continents. J Allergy Clin Immunol. (2014) 133:818–826 e814. doi: 10.1016/j.jaci.2013.09.038 24290283 PMC3969582

[51] Amenyogbe N Dimitriu P Cho P Ruck C Fortuno ES 3rd Cai B. Innate immune responses and gut microbiomes distinguish HIV-exposed from HIV-unexposed children in a population-specific manner. J Immunol. (2020) 205:2618–28. doi: 10.4049/jimmunol.2000040 PMC7653510 33067377

[52] Ling Y Jiang C Xiao Z Shang X Li Q Wang B. Serum adenosine deaminase activity and acute cerebral infarction: a retrospective case-control study based on 7913 participants. Aging (Albany NY). (2022) 14:8719–28. doi: 10.18632/aging.204338 PMC9699761 36260871

[53] Hirschhorn R Martiniuk F Rosen FS. Adenosine deaminase activity in normal tissues and tissues from a child with severe combined immunodeficiency and adenosine deaminase deficiency. Clin Immunol Immunopathol. (1978) 9:287–92. doi: 10.1016/0090-1229(78)90100-9 627115

[54] Cassani B Mirolo M Cattaneo F Benninghoff U Hershfield M Carlucci F. Altered intracellular and extracellular signaling leads to impaired T-cell functions in ADA-SCID patients. Blood. (2008) 111:4209–19. doi: 10.1182/blood-2007-05-092429 PMC2288726 18218852

