# Supplementary material for: Plasma adenosine deaminase-1 and -2 activities are lower at birth in Papua New Guinea than in The Gambia but converge over the first weeks of life
Source: Front Immunol. 2024 Sep 25;15:1425349. doi: 10.3389/fimmu.2024.1425349 (PMC11461337; doi:10.3389/fimmu.2024.1425349)
Supplement: Supplementary file 1 [file DataSheet1.zip › Figure S1.pdf]

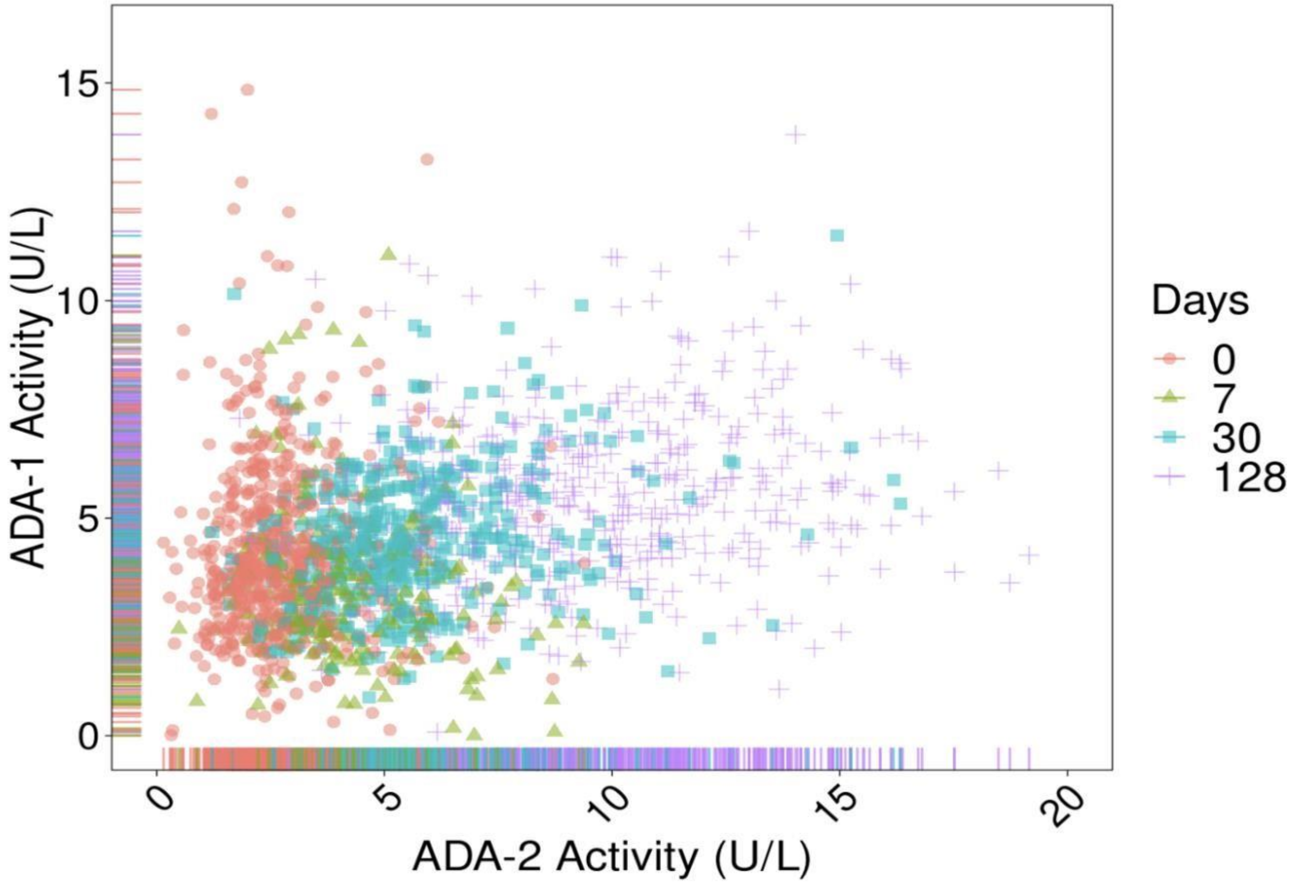

Figure S1: Scatterplot of plasma ADA-1 vs ADA-2 activity (in U/L) in infants in GAM across the first four months of life.
