# Supplementary material for: Plasma adenosine deaminase-1 and -2 activities are lower at birth in Papua New Guinea than in The Gambia but converge over the first weeks of life
Source: Front Immunol. 2024 Sep 25;15:1425349. doi: 10.3389/fimmu.2024.1425349 (PMC11461337; doi:10.3389/fimmu.2024.1425349)
Supplement: Supplementary file 1 [file DataSheet1.zip › Figure S3.pdf]

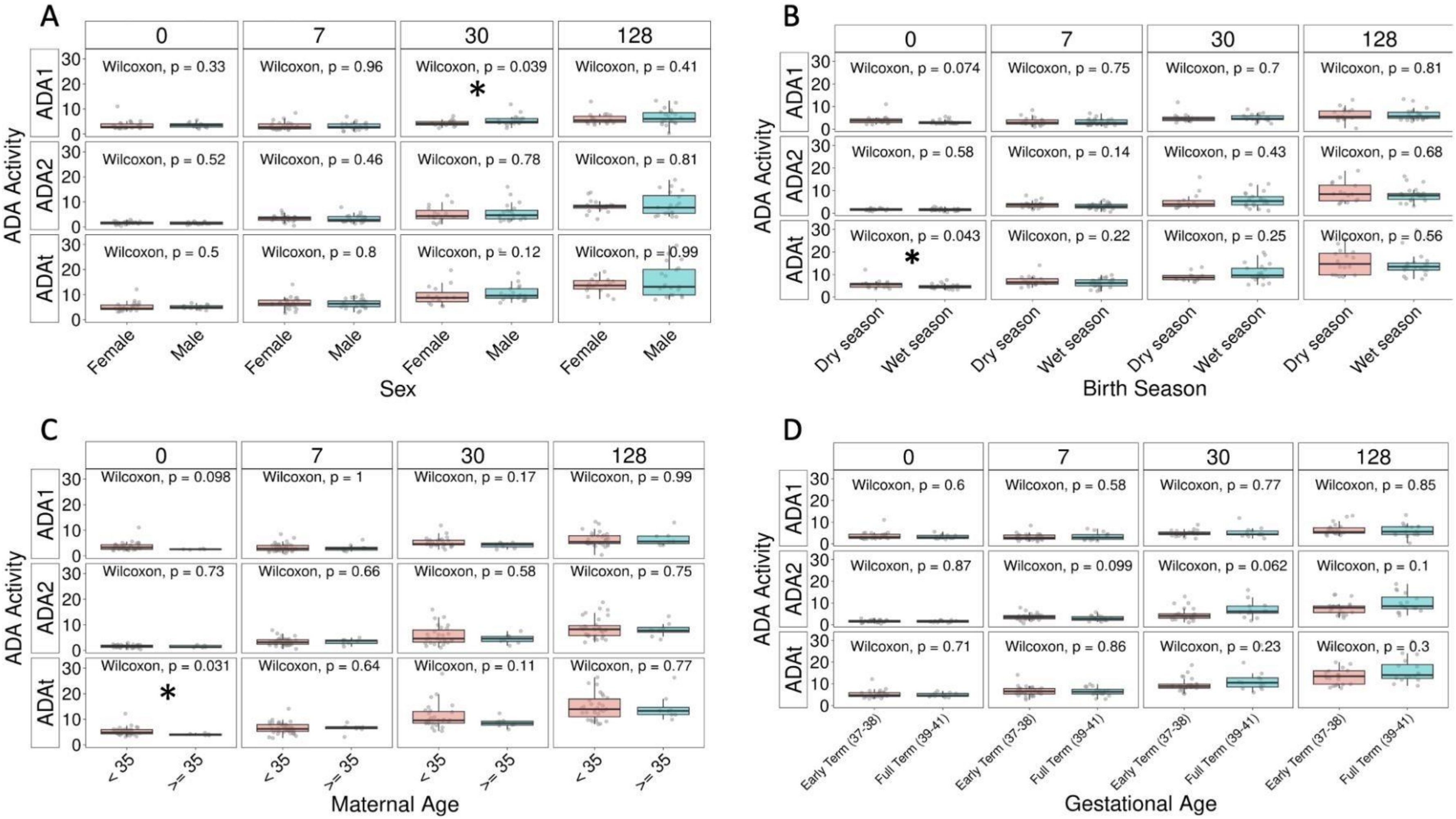

Figure S3: Comparison of ADA-1, ADA-2, and total ADA activity based on (A) sex, (B) birth season, (C) maternal age, and (D) gestational age in infants in PNG across the first four months of life.
