# Supplementary material for: Plasma adenosine deaminase-1 and -2 activities are lower at birth in Papua New Guinea than in The Gambia but converge over the first weeks of life
Source: Front Immunol. 2024 Sep 25;15:1425349. doi: 10.3389/fimmu.2024.1425349 (PMC11461337; doi:10.3389/fimmu.2024.1425349)
Supplement: Supplementary file 1 [file DataSheet1.zip › Table S1.pdf]

Table S1. Wilcoxon rank-sum test generated p-values between days grouped by ADA activity in PNG cohort (arranged from smallest to largest p-value)

ADA	Group 1 (Day)	Group 2 (Day)	p-value	Significance
ADA2	0	128	< 2e-16	****
ADAt	0	128	< 2e-16	****
ADAt	7	128	< 2e-16	****
ADA2	7	128	< 2e-16	****
ADAt	0	30	9.80E-15	****
ADA2	0	30	6.70E-14	****
ADA2	0	7	2.00E-13	****
ADA1	7	128	8.20E-12	****
ADA1	0	128	2.40E-10	****
ADAt	7	30	7.40E-09	****
ADA1	7	30	7.90E-08	****
ADA1	0	30	3.60E-06	****
ADAt	30	128	1.40E-05	****
ADAt	0	7	5.90E-05	****
ADA2	30	128	0.00011	***
ADA2	7	30	0.0006	***
ADA1	30	128	0.00548	**
ADA1	0	7	0.16917	ns
