# Supplementary material for: Plasma adenosine deaminase-1 and -2 activities are lower at birth in Papua New Guinea than in The Gambia but converge over the first weeks of life
Source: Front Immunol. 2024 Sep 25;15:1425349. doi: 10.3389/fimmu.2024.1425349 (PMC11461337; doi:10.3389/fimmu.2024.1425349)
Supplement: Supplementary file 1 [file DataSheet1.zip › Table S2.pdf]

Table S2. Wilcoxon rank-sum test generated p-values between days grouped by Sex in PNG cohort (arranged from smallest to largest p-value)

ADA	Day	Group 1 (Male)	Group 2 (Female)	p-value	Significance
ADA1	30	Male	Female	0.039	*
ADAt	30	Male	Female	0.122	ns
ADA1	0	Male	Female	0.327	ns
ADA1	128	Male	Female	0.411	ns
ADA2	7	Male	Female	0.457	ns
ADAt	0	Male	Female	0.499	ns
ADA2	0	Male	Female	0.518	ns
ADA2	30	Male	Female	0.78	ns
ADAt	7	Male	Female	0.804	ns
ADA2	128	Male	Female	0.813	ns
ADA1	7	Male	Female	0.96	ns
ADAt	128	Male	Female	0.989	ns
