# Supplementary material for: Plasma adenosine deaminase-1 and -2 activities are lower at birth in Papua New Guinea than in The Gambia but converge over the first weeks of life
Source: Front Immunol. 2024 Sep 25;15:1425349. doi: 10.3389/fimmu.2024.1425349 (PMC11461337; doi:10.3389/fimmu.2024.1425349)
Supplement: Supplementary file 1 [file DataSheet1.zip › Table S3.pdf]

Table S3. Wilcoxon rank-sum test generated p-values between days grouped by birth season in PNG cohort (arranged from smallest to largest p-value)

ADA	Day	Group 1 (Dry Season)	Group 2 (Wet Season)	p-value	Significance
ADAt	0	Dry season	Wet season	0.043	*
ADA1	0	Dry season	Wet season	0.074	ns
ADA2	7	Dry season	Wet season	0.141	ns
ADAt	7	Dry season	Wet season	0.221	ns
ADAt	30	Dry season	Wet season	0.249	ns
ADA2	30	Dry season	Wet season	0.434	ns
ADAt	128	Dry season	Wet season	0.563	ns
ADA2	0	Dry season	Wet season	0.582	ns
ADA2	128	Dry season	Wet season	0.682	ns
ADA1	30	Dry season	Wet season	0.702	ns
ADA1	7	Dry season	Wet season	0.755	ns
ADA1	128	Dry season	Wet season	0.81	ns
