# Supplementary material for: Plasma adenosine deaminase-1 and -2 activities are lower at birth in Papua New Guinea than in The Gambia but converge over the first weeks of life
Source: Front Immunol. 2024 Sep 25;15:1425349. doi: 10.3389/fimmu.2024.1425349 (PMC11461337; doi:10.3389/fimmu.2024.1425349)
Supplement: Supplementary file 1 [file DataSheet1.zip › Table S4.pdf]

Table S4. Wilcoxon rank-sum test generated p-values between days grouped by Maternal age in PNG cohort (arranged from smallest to largest p-value)

**ADA**	**Day**	**Group 1 (< 35)**	**Group 2 (>= 35)**	**p-value**	**Significance**
ADAt	0	< 35	>= 35	0.031	*
ADA1	0	< 35	>= 35	0.098	ns
ADAt	30	< 35	>= 35	0.113	ns
ADA1	30	< 35	>= 35	0.166	ns
ADA2	30	< 35	>= 35	0.576	ns
ADAt	7	< 35	>= 35	0.642	ns
ADA2	7	< 35	>= 35	0.661	ns
ADA2	0	< 35	>= 35	0.729	ns
ADA2	128	< 35	>= 35	0.746	ns
ADAt	128	< 35	>= 35	0.772	ns
ADA1	128	< 35	>= 35	0.986	ns
ADA1	7	< 35	>= 35	1	ns
